# House Mice as a Real Sanitary Threat of Human and Animal Leptospirosis: Proposal for Integrated Management

**DOI:** 10.1155/2019/3794876

**Published:** 2019-06-23

**Authors:** Aurélie Marquez, Tristan Ulivieri, Etienne Benoit, Angeli Kodjo, Virginie Lattard

**Affiliations:** ^1^USC 1233 RS2GP, VetAgro Sup, INRA, University of Lyon, 69280 Marcy-l'Etoile, France; ^2^Laboratoire des Leptospires, VetAgro Sup, 69280 Marcy-l'Etoile, France

## Abstract

Leptospirosis is a reemerging zoonosis and ranges in severity from benign to sometimes fatal. In cattle, infection may be responsible for abortion and infertility cases causing economic losses. Humans may be contaminated through direct contact with urine of infected animals or indirectly though interaction with urine-contaminated environment. Many wildlife species living close to cattle, especially commensal rodents may play a role in the transmission of leptospires. Because little is known on the epidemiology of nonmaintenance* Leptospira* serovars, appropriate management is still limited. On a French farm where human and cattle leptospirosis were detected, the transmission cycle was explored to propose appropriate mitigation measures. For that, commensal rodents present on the farm were trapped and their leptospires carriage was studied by molecular methods. Trapped mice were shown to carry two pathogenic* Leptospira* species (*L. interrogans* and* L. kirschneri*). Since these 2 serogroups were simultaneously detected in the trapped mice and in the cows of this farm, we suspected an initial* Leptospira* transmission from mice to cows requiring an effective management of mice on this farm. Because resistance to anticoagulant rodenticides due to* Vkorc1* mutations has been largely described in rodents and first-generation anticoagulant rodenticides seemed to be inefficient in controlling mice on this farm, susceptibility of these mice to anticoagulants has been characterized by* Vkorc1* sequencing. 50% of the trapped mice carried mutations in the* Vkorc1* gene leading to severe resistance to first-generation anticoagulants. The management of such mice that are a real sanitary threat can be achieved only by using the most toxic second-generation anticoagulants or nonanticoagulant solutions.

## 1. Introduction

Leptospirosis is a worldwide zoonosis caused by pathogenic leptospires, bacteria from the spirochete order [1, 2]. This disease is reemerging [3] affecting approximately 1 million people all over the world each year [4, 5]. Over the last few years, the number of cases has increased in tropical regions [6]. Leptospirosis ranges in severity from benign in most cases to sometimes fatal with a mortality rate of around 10 percent and raises a real public health issue [7]. This disease can provoke a large variety of symptoms. Renal and hepatic failures are observed in human cases [8]. For treatment of human infections, antibiotics as penicillin and doxycycline can be used. In cattle, abortions and other reproductive problems are observed [9] causing economic losses for farmers. The World Organization for Animal Health (OIE) recommends streptomycin for farm animals [10]. Vaccines against some serovars are also available, depending on the country. The main route of contamination is through indirect contact with infected urine from carriers. Main carriers are rodents like rats and coypus but small mammals as mustelids can participate in leptospirosis transmission [11, 12]. They accumulate bacteria in kidneys and excrete them by their urine. Excreted leptospires can thus survive in water and manure for several weeks [1, 3], which may provide recontamination pathways. Because little is known on the epidemiology of leptospires, appropriate management is still limited. Nevertheless, as many rodents are main carriers of leptospires, efficient rodent management could prevent leptospirosis transmission.

Rodent management may associate sanitary and architectural approaches as well as adapted farming practices. If insufficient, these approaches may be completed by the use of chemical rodenticides. Different classes of molecules are used in Europe (i.e., antivitamin K anticoagulants (AVKs), zinc phosphide, alpha-chloralose,* etc.*) with different usage restrictions. In France, AVKs are the main used rodenticides because of their delayed action avoiding food aversion. AVKs provoke haemorrhage by inhibiting the vitamin K epoxide reductase (VKORC1) involved in the vitamin K cycle limiting the activation of the vitamin K-dependent clotting factors II, VII, IX, and X.

Because of the intensive use of AVKs, resistance to AVKs has been reported since 1960 [13, 14], associated with* Vkorc1* mutations [15–18]. Since the first description, numerous mutations of* Vkorc1* have been reported for brown rats (*Rattus norvegicus*) and house mice (*Mus musculus*). To overcome resistance problems, a second-generation of AVKs including bromadiolone, difenacoum, brodifacoum, flocoumafen, and difethialone has been developed. This second-generation of AVKs is more toxic, more tissue-persistent, and thus more often associated with primary or secondary poisonings.

After the demonstration of animal and human leptospirosis cases in a cattle farm located in the Auvergne-Rhône-Alpes region of France, our study aims to identify the most likely transmission scheme(s) on this farm and the methods to be used to prevent any new cases.

## 2. Materials and Methods

### 2.1. Study Area

**Figure 1:**
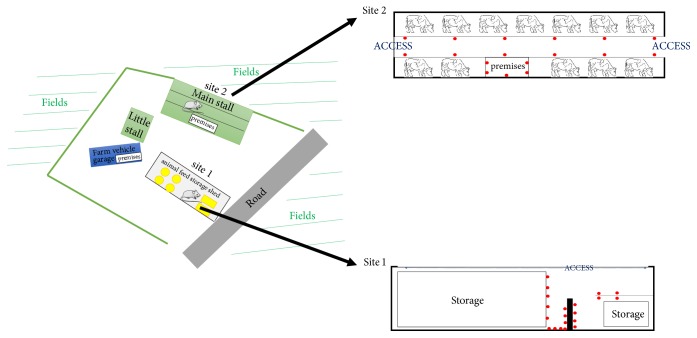
Farm scheme and trapping map.

This study was conducted on a farm in the Auvergne-Rhône-Alpes region of France, in the Loire department. This farm was located in lowland surrounded by hills, served by a single road and divided into different agricultural buildings (two stalls, one animal feed storage shed and one vehicle shed) (Figure 1). Two farmers were working on this farm to raise cattle for meat production. The herd was composed of 300 head of Charolais cattle with 130 adult cows. Animals were in the meadow and exclusively fed with grass in summer, and were in stabling and fed with hay and silage in winter. First-generation anticoagulant rodenticides were used by the farmer to control rodent infestation into agricultural buildings.

### 2.2. Rodent Trapping Method

Mice were trapped using 40 mouse traps (Aegis Trap Souris, De Sangosse, France), set up where traces of rodents had been observed (Figure 1) in the animal feed storage shed (referred to as site 1) and in the main stall (referred to as site 2). No rats and no trace of rats were observed in the different farm buildings. Baiting was done using peanut butter daily renewed during the capture period. Trapping was conducted during 24 consecutive days from November 17th, 2015, to December 10th, 2015. The trap effort (*i.e.*, number of traps per site × number of days of trapping) was 480 trap-days in sites 1 and 2, respectively.

During the capture period, cows were mainly housed in the barn. Trapped animals were weighted, sexed, and assigned to one of two age categories: immature house mice (< 12g) and mature house mice (> 12g). Dead mice were autopsied in the laboratory. Kidneys were taken under sterile conditions. One part of the kidney was used immediately for culture, the other part (*i.e.*, corticomedullary junction, 5 mg) was frozen at -20°C for the molecular detection and characterization of leptospires. A piece of tail of each rodent was stored in 70% ethanol as material for DNA extraction for* Vkorc1* genotyping.

### 2.3. Rodent Species Identification

Trapped animals were molecularly confirmed to be* M. musculus domesticus* (as opposed to a morphologically similar species) by sequencing a portion of the* mtDNA cytochrome b* gene. Briefly, genomic DNA was extracted from liver using silica columns (Macherey Nagel) according to the manufacturer's recommendations. Two microliters of genomic DNA were amplified by PCR using specific primers of* cytochrome b* [19]. The amplified product was sequenced on both strands; the resulting sequence was submitted to blast analysis (http://blast.ncbi.nlm.nih.gov).

### 2.4. Serological Tests Using the Microscopic Agglutination Test (MAT)

MAT analyses were performed in the Laboratoire des Leptospires (VetAgro Sup, Marcy-l'Etoile, France) as described by [20] using a total of 23* Leptospira* strains belonging to 14 serogroups. Blood samples were collected from seventy-six cows presenting reproductive disorders and centrifuged, and the serum obtained was stored at −20°C until MAT analyses. The screening was performed starting with a serum dilution of 1: 10 up to a dilution of 1: 800. The endpoint was the highest serum dilution showing 50% agglutination in free-moving leptospires. Positivity to a serovar was concluded when a MAT reaction was observed towards this unique serovar or when this serovar displayed a titer three times higher than the other serovars when cross reactions were observed. When cross-reactivities were observed between several serovars with similar titers, positivity was established towards cross-reacting serovars.

### 2.5. Leptospira Isolation

Kidney from each mouse and 3 placental cotyledons from aborted cows were crushed and aseptically transferred to tubes containing Ellinghausen McCullough Johnson and Harris (EMJH) medium (Indicia, St Génis, France) [20]. Three serial dilution tubes were incubated at 30°C according to the protocol for pathogenic* Leptospira* isolation [20, 21]. For a period of three months, the tubes were examined weekly using a dark-field microscope

### 2.6. 16S Amplification for* Leptospira* Detection and Species Identification

DNA extractions of mice kidneys were performed with QIAamp® DNA Mini Kit, Qiagen, according to manufacturer's guidelines. Amplification of a part of 16S rDNA was performed to detect* Leptospira* and identify species as previously described [21, 22] using LeptA primer 5'-GGCGGCGCGTCTTAAACATG-3' and LeptB primer 5'-TTCCCCCATTGAGCAAGATT-3'. PCR mix without the target DNA was included as a negative control, and PCR mix with a Leptospira strain was included as a positive control. PCR products were then sequenced by Genoscreen enterprise by a Sanger procedure. The resulting sequence was submitted to blast analysis ((http://blast.ncbi.nlm.nih.gov).

### 2.7. Multisequence Typing (MST) Analyses

Positive samples for* L. interrogans* species were subjected to MST analyses allowing the sequencing of 3 intergenic regions, the MST1, MST3, and MST9 regions [23]. PCR mix without the target DNA was included as a negative control, and PCR mix with a* Leptospira* strain was included as a positive control. PCR was negative for samples tested; therefore sequencing was not necessary.

### 2.8. Variable Number Tandem Repeat (VNTR) Analyses

Positive samples for* Leptospira sp.* by 16S amplification were subjected to VNTR analyses allowing us to determine the number of the repetition of short sequences located in three* Leptospira* genomic regions [24]. The following 3 regions were amplified: VNTR4, VNTR7, and VNTR10. DNAs from reference* Leptospira* strains were used as control and to determine VNTR loci profiles. The mix for PCR was prepared using 1x PCR buffer (50mM KCl, 10mM Tris-HCl [pH8.3], 3 mM MgCl2), 0.2mM dNTP, 0.2 mM of each primer (VNTR4F: 5'-AAGTAAAAGCGCTCCCAAGA-3', VNTR4R: 5'-ATAAAGGAAGCTCGGCGTTT-3', VNTR7F: 5'-GATGATCCCAGAGAGTACCG-3', VNTR7R: 5'-TCCCTCCACAGGTTGTCTTG-3', VNTR10F: 5'-GAGTTCAGAAGAGACAAAAGC-3', and VNTR10R: 5'-ACGTATCTTCATATTCTTTGCG-3', previously described by [21]), 5 units of HotStarTaq® DNA Polymerase and 5 *μ*l of extracted DNA template, and sterile water to have a final volume of 50 *μ*l. The following thermocycling program was used: a 15-minute enzyme activation step at 95°C, followed by 40 cycles of 95°C for 30 s, 54°C for 30 s (VNTR4 and VNTR7)/52°C for 30s (VNTR10), and 72°C for 1 min with a final elongation step at 72°C for 10 min. PCR mix without the target DNA was included as a negative control, and PCR mix with a* Leptospira* strain was included as a positive control and band size control for electrophoresis gel migration.

### 2.9. *Vkorc1* Genotyping

Genomic DNA extractions from mice tails were performed with QIAamp® DNA Mini Kit, Qiagen, according to manufacturer's guideline.* Vkorc1* gene was amplified as previously described [25]. The amplified products were sequenced on both strands. Obtained sequences were subsequently compared with the published* Vkorc1* gene sequences for* Mus musculus domesticus* (Genbank n° GQ905710.1).

### 2.10. Ethics Statement

This project was run in the framework of a rodent control program decided and organized by the farmer. Trapping was conducted by a private pest control firm during an eradication program conducted on the farm. Commercial mouse traps killing instantly mice were used. Traps were placed by the pest control operator where rodent tracks were detected. Daily control of the traps was done by the farmer. When the presence of rodent in the trap was noted by the farmer, the latter placed it at 4°C until its dissection the next day. This study was not considered to be an “experimental procedure” as defined by the French legislation (Rural Code, Article R214–89) and therefore was not subjected to an ethical committee approval in France. This study complied with the ethical standards of European regulations governing the care and use of animals in research and it did not involve any endangered or protected species, or protected areas.

## 3. Results

### 3.1. Rodent Infestation on Farm

A total of 12 house mice (*Mus musculus domesticus*) were caught, all in site 2 (about 2 every 2 days), and referred to as S1 to S12, including 100% of mature animals and 50% of males as shown in Table 1. The trap success (*i.e.*, number of trapped mice/trap effort × 100) was 2.5 in site 2.

Trapped individuals were confirmed to be* Mus musculus domesticus* based on matching of mtDNA cytochrome b sequences. Amplified sequences presented more than 98% of homology with sequences of* cytochrome b* published for* Mus musculus domesticus.*

### 3.2. Leptospiral Carriage in Cows

Seventy-six cows presenting reproductive disorders and three aborted cows were subjected to serological analysis by MAT test. Reactivity towards the different tested serogroups with titers between 10 and 640 is presented in Table 2.

Among cows with reproductive disorders, forty-three were seropositive to a single serotype, either* L. Sejroe* (serovar Hardjo) or* L. Grippotyphosa* (Table 3) and twenty-three presented cross-reactivity against* Leptospira*.* L. Sejroe* (serovar Hardjo),* L. Grippotyphosa*, and to a lesser extent* L. Icterohaemorrhagiae*.

Out of the 3 aborted cows, 2 and 1 were demonstrated by MAT analysis to be infected by* L. Sejroe* and* L. Grippotyphosa*, respectively (Table 2). Unfortunately, all the attempts of cultures and PCR detection on aborted materials were unsuccessful.

### 3.3. Leptospiral Carriages in Trapped Mice

Based on 16S amplification, 7 mice presented positive results for* Leptospira* presence. Three* Leptospira* genospecies were identified, including a saprophytic leptospire.* L. biflexa* was found in 2 mice,* L. kirschneri* in 3 mice, and* L. interrogans* in 1 mouse. One mouse was found carrying the two leptospires species* L. interrogans* and* L. kirschneri* (Table 1). The prevalence of pathogen leptospiral renal carriage in trapped mice was 42%.

From the* L. interrogans* DNA sample extracted, MST and VNTR profiles could not be obtained because of unspecific amplifications. From the 4* L. kirschneri* DNA samples extracted, complete VNTR profiles were obtained in 2 samples and corresponded to* Leptospira* belonging to serogroup* Icterohaemorrhagiae* as shown in Table 1 according to VNTR published database [24]. For the two other samples, VNTR profiles were incomplete because VNTR10 analyses could not be interpreted because of the presence of unspecific bands.

### 3.4. *Vkorc1* Genotyping Results

In the trapped mice, 6 different mutations were found either alone or in combination. Mutations found in the trapped mice are summarized in Table 1. In exon 1, 4 mutations were detected. These mutations were located at nucleotide 34 (g.34C>T), 76 (g.76G>T), 111 (g.111A>G), and 142 (g.142G>C). Except for the g.111A>G mutation, these mutations were missense mutations leading to mutations R12W, A26S, and A48T, respectively. In exon 2, one missense mutation was detected at nucleotide 976 (g.976G>T) leading to mutation R61L. In exon 3, one missense mutation was detected at nucleotide 2223 (g.2223A>G) leading to mutation Y139C.

Four different* Vkorc1* genotypes were observed in the trapped mice. One genotype with no* Vkorc1* mutation was detected in 6 mice. One genotype with only 1* Vkorc1* mutation (g.2223A>G) leading to the Y139C protein mutation was detected in 3 mice. One genotype combining 2 missense mutations (g.34C>T and g.76G>T) leading to the R12W and A26S protein mutations and one silent mutation (g.111A>G) was found in only one mouse at the heterozygous state. The last genotype combining 4 missense mutations (g.34C>T, g.76G>T, g.142G>A, and g.976G>T) leading, respectively, to the R12W, A26S, A48T, and R61L protein mutations and one silent mutation (g.111A>G) was found in two mice, one at the homozygous state and one at the heterozygous state.

## 4. Discussion

### 4.1. Epidemiology of Nonmaintenance Leptospira Serovars on This Farm

The high rate of abortion observed in the herd, whereas in previous years nothing abnormal had been reported, the detection of antibodies against leptospires in 66 cows out of 76 tested, and the fact that the farmer contracted leptospirosis at the same time allowed us to suspect the recent establishment of a leptospires transmission cycle on this farm.

Three serogroups,* L. Sejroe* (serovar Hardjo),* L. Grippotyphosa*, and to a lesser extent* L. Icterohaemorrhagiae*, were found in cows by MAT analyses. The three aborted cows were found to be infected either by* L. Sejroe* for two cows or by* L. Grippotyphosa* for the other one. These results suggest that both serogroups are responsible for abortions observed in this herd. Since cows are known to be natural hosts for serovar Hardjo [9, 26] and the spirochete can survive in cow for years, it seems unlikely that the high rate of abortion and reproductive disorders observed at the time of the study was caused by the serovar Hardjo. Indeed, we can assume that in the absence of new animals entering the farm, the cows of this farm were already carriers of* L. Sejroe* (serovar* L. Hardjo*) in the previous years, while the reproductions proceeded normally. It is more likely that the high rate of abortions and the reproductive disorders suddenly observed was due to recent infection by the other serogroups* L. Grippotyphosa* and* L. Icterohaemorrhagiae* that are nonmaintenance* Leptospira* serovars in cows.

Many wildlife species living in the cattle habitats may be suspected to have a role in the initiation of* Leptospira* transmission cycle. Nevertheless, since rodents are the natural carriers of these serogroups [27, 28], it was normal to immediately consider the role of commensal rodents in this initiation even if rodent control operations have been regularly conducted by the farmer. The presence of rats has not been highlighted in the farm buildings despite a careful inspection, which seems to show the success of the solutions applied by the farmer to control rat infestations. On the contrary, trace of mice and mice themselves have been seen in two buildings of the farm, suggesting a failure of the solutions applied to control mice. Only 12 mice could be captured in site 2, while numerous mice and numerous traces of mice could be observed in sites 1 and 2. The strong food competition in the buildings has undoubtedly limited the success of trapping, preventing any conclusion on the size of the mice populations present on this farm. Nevertheless, our study highlights the presence of mice carrying two pathogenic* Leptospira* species (*L. interrogans* and* L. kirschneri*) (5/12 trapped mice) in the main stall of the farm. Within these 2* Leptospira* species, 3 serovars were identified, one completely identified (*i.e.*, Icterohaemorrhagiae) and the other two partially identified. We assume that the DNA of the* L. kirschneri*, partially typed by VNTR method, belongs to* Grippotyphosa* serogroup, the major representative of the* L. kirschneri* species in France, for which VNTR analysis has shown a weak ability to achieve its typing [29].

**Figure 2:**
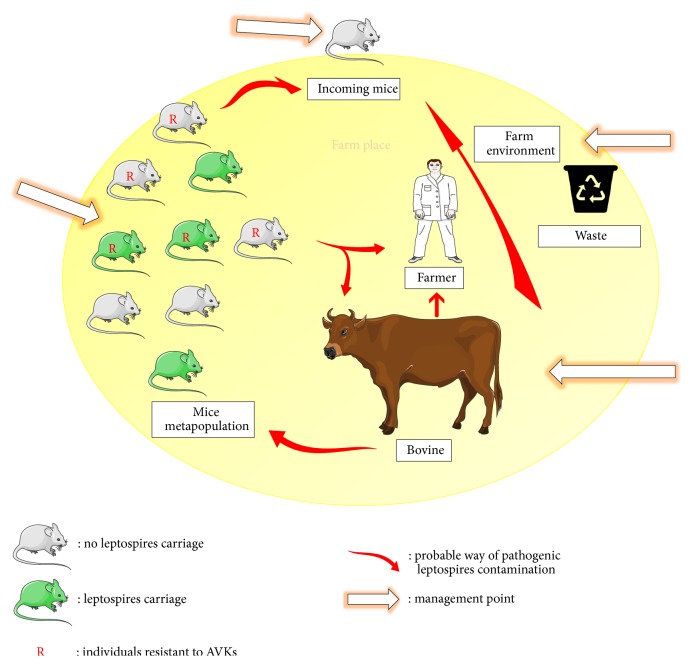
Leptospira transmission cycle (represented by the red arrows) and points of management (represented by the white arrows).

Since these 2 serogroups (*Grippotyphosa and Icterohaemorrhagiae*) were simultaneously detected in mice and cows, we could suspect an initial* Leptospira* transmission from mice to cows and then, hypothetically, from cows to the farmer who developed the disease at the period of cow abortions (Figure 2). Because mice share the same habitat as cows on this farm, initial transmission from mice to cows was most likely indirect through interaction with a urine-contaminated environment such as hay and grains. Indeed, contact between rodents and food in dairy cattle has been often described as a factor risk in a prevalence of* Leptospira* infection study [9, 29]. After the initial transmission from mice to cows, we can hypothesize that the farmer acquired leptospirosis from cow through direct contact with the urine of infected animals or indirectly with a urine-contaminated environment such as manure. Even if the serogroup of* Leptospira* responsible for leptospirosis of the farmer is unknown, we can easily assume that the serogroup* Grippotyphosa* was also responsible for the leptospirosis of the farmer. In fact, in a recent epidemiologic study on human leptospirosis performed in the Franche-Comté department, located approximately 300 km at the North East of the farm, this serogroup was found to be the most often involved in human disease [8].

This farm, in which animal leptospirosis is concomitant to human leptospirosis, is clearly a public health concern. Indeed, leptospirosis is reported as fatal in about 10% of infected humans, with a possible fatality range of 5–40%. The context of this farm clearly shows that the importance of these 2 nonmaintenance serovars in bovine leptospirosis should not be underestimated in epidemiological survey studies. Radical measures must be implemented within the farm to stop the cycle. Different possible management points will be discussed above to limit and prevent* Leptospira* transmission.

### 4.2. Management Implications

*Management of mice in buildings*: Until now, scientific studies have mainly shown that* Leptospira* is frequently carried in rats and studies reporting carriage of* Leptospira* in mice are few [30]. This could explain why the farmer was not worried about the presence of mice in these buildings. Because leptospiral-infected mice share the same habitat as cows on this farm, they represent a real threat even if infestation was limited. The first action to be implemented is to effectively manage rodents in livestock buildings, but also in food and materials storages. The farmer had applied first-generation anticoagulants. Clearly this method has been effective only in controlling rats. The genetic analysis carried out on the trapped mice enabled us to understand the failure of this treatment. In fact, 50% of the trapped mice carried mutations in the* Vkorc1* gene. Mutations of this gene have been widely studied in Europe and France because a number of them have been shown to be responsible for resistance to AVKs which are the most frequently used molecules in Europe to manage rodents. The mutations found on this farm have already been described in Europe and France [31, 32]. In the South East of France, its frequency has been reported to be close to 20% [32] in mice trapped from 21 different sites. It is therefore not surprising to find this mutation in this site. The set of mutations (R12W, A26S, A48T, R61L) detected on this farm has also been previously described in Germany [31, 33] and then in France [25, 32]. This set of 3 or 4 mutations has been reported to be the consequence of the introgression of* Vkorc1* gene from the Algerian mouse (*Mus spretus*) in the genome of* Mus musculus domesticus*. The Y139C mutation and the set of 3 or 4 mutations have been shown to be responsible for severe resistance to anti-vitamin K anticoagulants, particularly to first-generation molecules. The failure of the treatments carried out by the farmer is therefore understandable, especially since this resistance is compounded by the presence in the buildings of easily accessible food stocks and certainly responsible for a low consumption of baits by mice. The management of such mice can be achieved only by using second-generation anticoagulants such as difenacoum, difethialone, brodifacoum, or flocoumafen. These anticoagulants are more effective in such strains, but they are more persistent and more often associated with secondary poisoning of wildlife due to the ingestion of intoxicated rodents. Their use must therefore be limited to the area around a building and it is better to use them in baiting boxes.

*Limiting access to incoming mice*: The presence of several leptospiral strains and several mutations of the* Vkorc1* gene within the same farm is quite surprising. Indeed, generally within the same site and the same rodent family, a single leptospiral strain or a unique* Vkorc1* gene mutation is found. For example, a recent study conducted in the city of Lyon (France) showed the presence of a unique strain of leptospires in rats caught in different sites of the same city [22]. Similarly, a study conducted in a Paris park of approximately 80 ha reported the presence of a unique* Vkorc1* gene mutation in a sample of more than 80 rats trapped at different park locations [34]. On this farm, 2 species and at least 3 leptospires serovars were detected with individuals potentially carrying 2 species of leptospires, as well as 4 different* Vkorc1* genotypes. Two hypotheses are possible: (1) Within the farm several micropopulations of mice forming a metapopulation could be present, nesting in different places of the farm/building and crossing very rarely, possibly in places where they eat, leading potentially to a transfer of pathogens or even resistance alleles; a suitable management as explained previously must be done by considering all the potential sites of breeding and feeding. (2) Within the farm a single micropopulation is present, and the diversity in pathogens and* Vkorc*1 genotypes is due to new entrants. Transportation of calves, slaughter, and feedstuffs certainly fosters the exchange of mice by transportation, which can result in the variety of genotypes and L. species/serovars in one location. New entrants introduced by transport or from the surrounding area could exchange genes and pathogens with local individuals when animals meet; control measures should be combined with architectural and sanitary measures to prevent the introduction of new entrants or the dispersion of outward bounds. Indeed, the presence of rodents carrying pathogenic leptospires in the farm poses a risk for all stakeholders exchanging products with this farm. Nevertheless, on a farm, control measures are extremely difficult or impossible to implement. Moreover, the plurality of* Vkorc1* gene mutations found in a homozygous state on the same site could favor recombination between genotypes that could generate resistance to the first-generation AVKs, but also to the second-generation AVKs as already described by [32].

*Prophylaxis measures*: On this farm, to reduce the risk of pathogenic leptospires contamination, prophylaxis measures are also important in order to reduce farm and domestic animals and human contamination, but also contamination of new entrant mice. Containment of seeds to prevent them to be contaminated by infected mice urine and disinfection of equipment and surfaces which could have been in contact with the pathogen must be done as far as possible. If possible calving boxes and quarantine zones must be set up for cattle, before spreading manure in pasture, a storage period higher than 8 days should limit risk of contamination because of the short survival period of leptospires in manure [35].

Human vaccine could be preconised against serovar* Icterohaemorrhagiae*. The infecting strain of the farmer could not be characterized, but symptoms were febrile illness associated with mild hepatitis and this form is usually known to be caused by* Grippotyphosa*, a serovar considered to be less severe than clinical forms caused by* Icterohaemorrhagiae*. In France, only vaccine against serovar* Hardjo* is available for bovine. In this case, using this vaccine solely would not be efficient unless it is associated with* Grippotyphosa* antigen as the vaccines available in the US. Our study points out that* Hardjo* is not the only serovar responsible for bovine abortion and the vaccine to be used should therefore associate antigens other than the only* Hardjo* antigen which is currently available for controlling bovine leptospirosis in France.

## 5. Conclusions

Reproductive disorders and abortions suddenly observed on this farm could be associated with leptospirosis. Since L*. Sejroe* (serovar Hardjo),* L. Grippotyphosa*, and* L. Icterohaemorrhagiae* have been detected in cows, clinical symptoms may be due to one or more of these serogroups and appropriate measures should be taken. The presence of* L. Sejroe* (serovar Hardjo) would justify the vaccination of the herd against this serovar. However, this vaccination will not prevent contaminations of cows by* L. Grippotyphosa* or* L. Icterohaemorrhagiae* carried by mice that could also contribute to clinical signs observed on this farm.

The fact that several mice present on this farm carried* L. Grippotyphosa* or* L. Icterohaemorrhagiae* demonstrates the relevance of mice control on this farm to mitigate the risk of* Leptospira* transmission from wildlife to livestock and/or humans even if the number of mice captured was limited. Nevertheless, management of such mice carrying zoonotic agent is challenging because of their severe resistance to AVK rodenticides. The low sampling of captured mice did not allow us to compare the prevalence of leptospiral carriage between resistant mice and nonresistant mice. A more complete study should be conducted to evaluate a potential link between resistance and carriage of leptospires. A biological cost of resistance could possibly lead to a more frequent contamination of resistant mice. Once the transmission cycle is established in livestock, rodent control must be combined with appropriate environmental and medical prophylactic measures. It would have been interesting to be able to repeat MAT analysis after the implementation of all the recommended measures to know the evolution of the situation, but unfortunately this was not possible.

Such surveillance studies are essential to identify circulating serovars that are essential for the development of appropriate vaccines. Because of the small number of trapped and analyzed mice, this study may be considered as a preliminary report on* Leptospira* carriage by house mice. More studies will be needed to assess* Leptospira* prevalence in mice.

## Figures and Tables

**Table 1:** Mice leptospires carriage and *Vkorc1* mutations.

Mice	Sex	Leptospires carriage		VKORC1 mutations			Resistance to AVK as reported by [24]
		Species	Serogroup	Exon 1	Exon 2	Exon 3	
S1	Male	*L. Interrogans & kirschneri*	Grippotyphosa^*∗*^	WT	WT	Y139C^*∗∗∗*^	+++
S2	Male	*L. kirschneri*	Icterohaemorrhagiae	WT	WT	Y139C^*∗∗∗*^	+++
S3	Male	X	X	WT	WT	WT	-
S4	Female	X	X	R12W/A26S/E37E^*∗∗*^	WT	WT	+
S5	Female	*L. kirschneri*	Grippotyphosa^*∗*^	R12W/A26S/E37E/A48T^*∗∗∗*^	R61L^*∗∗∗*^	WT	+++
S6	Male	X	X	WT	WT	WT	-
S7	Female	X	X	WT	WT	WT	-
S8	Female	X	X	WT	WT	WT	-
S9	Female	*L. interrogans*	Failed analyses	WT	WT	Y139C^*∗∗∗*^	+++
S10	Male	*L. biflexa*	ND	WT	WT	WT	-
S11	Male	*L. kirschneri*	Icterohaemorrhagiae	WT	WT	WT	-
S12	Female	*L. biflexa*	ND	R12W/A26S/A48T^*∗∗*^	R61L^*∗∗*^	WT	+ to +++

^*∗*^Presumptive serogroup; ^*∗∗*^heterozygous mutation; ^*∗∗∗*^homozygous mutation; WT: wild type; X: no leptospiral carriage found; ND: not done. Level of resistance induced by specific Vkorc1 genotype was previously reported by [25] and is indicated in this table as -, no AVKs resistance; +, limited AVKs resistance; and +++, severe AVKs resistance.

**Table 2:** MAT results of the samples taken from pregnant and aborted cows.

Tested serogroups	Number of positive serums among the 76 pregnant cows^*∗*^	MAT titres (min to max)^*∗∗*^	Number of positive serums in aborted cows (3 individuals)	MAT titres (min to max)
Icterohaemorrhagiae	21	10 -> 80	0	/
Australis	27	10 -> 80	0	/
Autumnalis	0	/	0	/
Ballum	0	/	0	/
Bataviae	0	/	0	/
Canicola	0	/	0	/
Grippotyphosa	10	20 -> 80	1	80
Hebdomadis	0	/	0	/
Panama	0	/	0	/
Pomona	0	/	0	/
Pyogenes	0	/	0	/
Sejroe	61	10 -> 640	2	10 -> 160
Tarassovi	0	/	0	/
Cynopteri	0	/	0	/

^*∗*^See next for detailed results. ^*∗∗*^ Positivity is recorded on the panel of serovars within the serogroup (i.e., within the Grippotyphosa serogroup, serovars Grippotyphosa and vanderhoedoni were tested; see text).

**Table 3:** Detailed results obtained for the MAT analysis of the 76 serums sampled from the pregnant cows.

Number of analyzed serum samples	*Number of serum samples with positivity to one or more serogroups*		
76 cattles	66 (87%)		
	*Detailed results of positivity*		
	Serogroup	Positivity ascertained to a single serotype^*∗*^	Cross reactions^*∗∗*^
	AUS	0	23
	GRIPP	1	
	IH	0	
	SJ	42	
	Total	43	23
		66	

^*∗*^ Positivity is ascertained when a MAT reaction is observed solely to this serovar or when this serovar displays a titer three times higher than the other serovars when cross reactions is observed. ^*∗∗*^ Cross reactions in which the previous parameters are not observed (in all these reactions, MAT titer against a member of the Sejroe serogroup was common). AUS: *australis*, GRIPP: *grippotyphosa*, IH: *icterohaemorrhagiae*, SJ: *sejroe*.

## Data Availability

All the data used to support the findings of this study are included within the article.
